# Dermal mesenchymal stem cells: a resource of migration-associated function in psoriasis?

**DOI:** 10.1186/s13287-019-1159-3

**Published:** 2019-02-13

**Authors:** Xuping Niu, Junqing Li, Xincheng Zhao, Qiang Wang, Gang Wang, Ruixia Hou, Xinhua Li, Peng An, Guohua Yin, Kaiming Zhang

**Affiliations:** 1 Shanxi Key Laboratory of Stem Cell for Immunological Dermatosis, Institute of Dermatology, Taiyuan City Centre Hospital, No. 1 Dong San Dao Xiang, Jiefang Road, Taiyuan, 030009 Shanxi Province China; 2 Department of Dermatology, Xijing Hospital, Fourth Military Medical University, No. 15 Changle Road West, Xi’an, 710032 Shanxi Province China

**Keywords:** Psoriasis, Mesenchymal stem cells, Migration, Quantitative real-time reverse transcription PCR, Western blot

## Abstract

**Background:**

Psoriasis is a chronic and systemic, immune-mediated, inflammatory disease. Mesenchymal stem cells have effects on the inflammatory microenvironment, including regulating the proliferation, differentiation, recruitment, and migration of immunocytes.

**Methods:**

To investigate whether dermal mesenchymal stem cells (DMSCs) may act on migration of immunocytes in psoriasis patients, 22 patients with psoriasis and 22 matching healthy controls (age and sex in this study) were recruited. Seven migration-associated genes including chemokine like receptor-1 (*CMKLR-1*), collagen type VIII alpha1 (*COL8A-1*), neuropilin and tolloid-like 2 (*NETO-2*), nik-related kinase (*NRK*), secreted frizzled-related protein (*SFRP*), sulfate 6-*O*-endosulfatase 2 (*SULF-2)*, and synaptotagmin-like protein 2 (*SYTL-2*) were analyzed by quantitative real-time reverse transcription PCR and western blot. Peripheral blood-derived mononuclear cells (PBMCs) migration to MSCs was measured using a Thanswell chamber system.

**Results:**

We observed the upregulation of *CMKLR-1*, *COL8A-1*, *NETO-2*, *NRK*, *SYTL-2*, and *SULF-2* in dermal mesenchymal stem cells derived from patients with psoriasis at both mRNA and protein level, however, a significant downregulation of *SFRP-2* between two groups. By contrast, there were no significant between-group differences at the mRNA and protein expression level of *NETO-2* and *SULF-2*. The migration assay showed that in vitro the normal PBMC migration to psoriatic DMSC group was a 6.3 ± 0.7-fold increase compared with the control group.

**Conclusions:**

The results may suggest a potential pathogenetic involvement of DMSCs on migration of monocytes in psoriasis. Immune responses are regulated at the level of DMSCs, which probably represent the cells primarily involved in the “psoriatic march.”

**Electronic supplementary material:**

The online version of this article (10.1186/s13287-019-1159-3) contains supplementary material, which is available to authorized users.

## Background

Psoriasis is one of the most common immune-mediated chronic inflammatory skin disorders. The pathogenesis of psoriasis is represented by T cell–mediated immune responses with complex cellular networks, including macrophages, keratinocytes, and T and B cells [1]. Ample evidence suggests that the dysregulation of immune cells in the skin, particularly T cells, plays a critical role in psoriasis development. Earlier studies indicated that in psoriasis, there was a reduction in the numbers of natural killer (NK) cells in peripheral blood, and these cells were present in plaques instead. Peripheral blood monocytes seem to be of importance in the initiation and maintenance of cutaneous inflammatory disorders such as psoriasis and atopic dermatitis [2]. NK cells have been implicated in the pathogenesis of psoriasis by virtue of their migration to the inflammatory site from the plaques [3]. However, the exact mechanism remains unclear.

Mesenchymal stem cells (MSCs) are progenitor cells with the capacity of self-renewal, multilineage differentiation, and immunomodulation. Strikingly, emerging experimental and clinical evidence has shown that systemic infusion of MSCs has significant immunosuppressive effects on the treatment of a variety of inflammatory and autoimmune diseases by inhibiting the proliferation and function of immune cells, including T cells, B cells, NK cells, monocytes/macrophages, dendritic cells (DCs), and neutrophils [4]. The immunomodulatory properties of MSCs are associated with direct cell–cell contact with T cells and/or the production of a variety of cytokines and chemokines. The cytokines and chemokines contribute to the consequent regulation of proinflammatory immune cells and the balance on the host immune homeostasis [5]. The multiple effects of MSCs on cellular immunity may reflect their diverse influences on the different T cell effector subpopulations and their capacity to specifically protect or induce Treg populations. MSC administration has also been shown to be variably associated with beneficial effects in autoimmune and transplant models, as well as in several human clinical trials [6]. MSCs have the potential to directly or indirectly inhibit disease-associated help T cells (Th1), Th2, and regulatory T cells (Tregs), as well as CD8^+^ lymphocytes, but many key questions regarding the potency, specificity, and mechanistic basis remain unanswered. Of note, but still subject for debate, is the capability of MSCs to inhibit T cell activation and proliferation and to affect T cell migration, favoring Treg phenotypes by several mechanisms [7]. Adhesion molecules are the most crucial players mediating the binding of leukocytes to the vascular endothelium and leading to their extravasations. MSCs may modulate the capability of activated lymphocytes by selectively acting on specific adhesion molecules and chemokine receptors involved in the extravasations and migration of activated T cells.

In one of earlier studies, we found that dermal mesenchymal stem cells (DMSCs) derived from patients with psoriasis had an imbalance in cytokine secretion, including the increased levels of epidermal growth factor (EGF), stem cell factor (SCF), and interleukin-1 (IL-1) as well as the decreased levels of basic fibroblast growth factor (bFGF), IL-3, IL-6, IL-8, and hepatocyte growth factor (HGF) [8]. There is evidence that MSCs can respond to chemotactic signaling molecules. Chemokines are small, chemoattractant cytokines that play a key role in the recruitment of leukocytes to sites of inflammation and injury. In addition, soluble proinflammatory factors released by activated lymphocytes strongly influence MSC modulatory activity, supporting the idea of an intense cross-talk between MSCs and immune cells. MSCs are known to secrete many cytokines and therefore the authors hypothesized that MSCs might offer an easily available way to deliver these chemokines therapeutically. The main aim of this study was to investigate whether MSCs can induce peripheral blood-derived mononuclear cell (PBMC) migration. To experimentally test this hypothesis, we analyzed seven migration-associated genes in the present study, including chemokine-like receptor-1 (*CMKLR-1*), collagen type VIII alpha1a (*COL8A-1*), neuropilin and tolloid-like 2 (*NETO-2*), nik-related kinase (*NRK*), secreted frizzled-related protein (*SFRP*), sulfate 6-*O*-endosulfatase 2 (*SULF-2)*, and synaptotagmin-like protein 2 (*SYTL-2*) in mesenchymal stem cells derived from psoriatic lesions.

## Materials and methods

### Specimen source

Twenty-two Han Chinese volunteers (13 women, 9 men; mean age, 30.9 years) who agreed to undergo plastic surgery were enrolled in this study. The volunteers showed no systemic or autoimmune diseases. We also recruited 22 Han Chinese patients (12 women, 10 men; mean age 35.2 years) with psoriasis vulgaris from our outpatient department, diagnosed based on both clinical symptoms and pathological features (see Additional file 1). None of the patients had ever been treated with immunomodulators or had a relevant family history. The patients were free of any systemic therapy for at least 4 weeks and did not use any topical therapy in the last 2 weeks.

### Culture and expansion of DMSCs

DMSCs were isolated, cultured, and characterized in accordance with our previous report [9]. DMSCs cultured to three passages were collected and used for quantitative real-time reverse transcription PCR (qRT-PCR) and western blotting assays.

### Flow cytometry analysis

DMSCs at the third passage were stained with fluorescein isothiocyanate (FITC)-conjugated or phycoerythrin (PE)-conjugated antibodies against CD14, CD29, CD34, CD44, CD45, CD73, CD90, or CD105. Thereafter, the surface markers of DMSCs were analyzed according to our previous researches [9].

### Differentiation assay

Passage 3 DMSCs were differentiated into osteoblast, adipogenic, and chondrogenic cells in Dulbecco’s modified Eagle’s medium (DMEM), Ham F12 (DMEM/Ham F12) medium with 10% fetal bovine serum (FBS), and additional supplementation of differentiation required according to our previous experiments [9].

### RNA extraction and cDNA synthesis

Total RNA was extracted using Trizol (Invitrogen, Carlsbad, CA, USA) and treated with DNase (Invitrogen). The purity was analyzed using an Agilent 2100 Bioanalyzer (Agilent Technologies, Palo Alto, CA, USA). RNA was purified using oligo (DT)-conjugated magnetic beads (Invitrogen), and used to synthesize cDNA (Takara, Shiga, Japan).

### Primer design and qRT-PCR

The relative expression levels of seven genes (*CMKLR-1*, *COL8A-1*, *NETO-2*, *NRK*, *SFRP-2*, *SULF-2*, and *SYTL-2)* were compared to that of the reference gene *ACTB* actin beta (a housekeeping gene) by qRT-PCR. cDNA from each group was used for qRT-PCR (ABI) in 20 μl; reactions containing 2 μl cDNA, 10 μl Premix EX Taq II Buffer Master Mix (Takara), 0.4 μl ROX reference dye (Takara), and 0.2 μl primers (BGI, Shenzhen, China). PCR was performed as follows: 30 s at 95 °C and 40 cycles of 5 s at 95 °C and 30 s at the appropriate annealing temperature. Melting curves were obtained between 60 and 95 °C with a ramp rate of 0.2 °C/s. PCR products were identified by 2% agarose gel electrophoresis.

The data presented were normalized to *ACTB* mRNA. To determine the relative mRNA expression levels, we used the delta-delta Ct method [10]. All primer sequences were shown in Table 1.

**Table 1:** Primer information in the qRT-PCR assay

Target gene	Primer sequence (5′-3′)		Annealing temperature (°C)	Product size (bp)
*ACTB*	Sense	AGAGCTACGAGCTGCCTGAC	60	270
	Antisense	AGCACTGTGTTGGCGTACAG		
*SFRP2*	Sense	ACGGCATCGAATACCAGAACA	58	176
	Antisense	CTCGTCTAGGTCATCGAGGCA		
*NETO2*	Sense	AGATGGGCCATTTGGTTTCTC	56	139
	Antisense	TGCTCGAAATCCCAGTCCTTC		
*SULF2*	Sense	CACTGGCAAGTACGTCCACAA	60	111
	Antisense	CTATTGAGGTACACGGCAAAGG		
*COL8A1*	Sense	GGGAGTGCTGCTTACCATTTC	56	75
	Antisense	AGCGGCTTGATCCCATAGTAG		
*NRK*	Sense	CATTGGCCTTGGTACTTATGGC	59	93
	Antisense	GTCTTACGAGCGTTCATCACTT		
*SYTL2*	Sense	GCCCAGTGTAAGGACTTAGCA	60	87
	Antisense	GCCTTTGTCTGGTAGCAAATAGG		
*CMKLR1*	Sense	CAGTTACGGTGATGAATACCCTG	60	115
	Antisense	GACGATGCTGTAGACCACCAC		

*bp* base pair

### Western blot assay

Protein lysates from passage 3 DMSCs (2 × 10^6^/ml) were analyzed by sodium dodecyl sulfate-polyacrylamide gel electrophoresis (SDS-PAGE) and transferred to a polyvinylidene fluoride membrane. Nonspecific binding was blocked with 5% skimmed milk in Tris-buffered saline containing 0.1% Tween 20 for 1 h at room temperature. The filter was incubated overnight at 4 °C with primary mouse or rabbit polyclonal antibodies (all from Abcam, UK) to human glyceraldehyde-phosphate dehydrogenase (GAPDH), *CMKLR-1*, *COL8A-1*, *NETO-2*, *NRK*, *SFRP-2*, *SULF-2*, and *SYTL-2*. The horseradish peroxidase-conjugated secondary antibody (anti-rabbit or anti-mouse) was diluted 1:5000 and incubated for 1 h at room temperature. The bands were detected using enhanced chemiluminescence reagent (Applygen Technologies Inc., Beijing, China). Densitometric quantification of the bands was performed on scanned images (FluorChemQ; ProteinSimple Inc., CA, USA). GAPDH was used as internal standard, and all protein levels were normalized to GAPDH (target protein value/GAPDH value).

### PBMC isolation

After obtaining informed consent, peripheral blood was obtained from healthy volunteers ranging in age from 18 to 35 years (four female and eight male donors). The mononuclear fractions (PBMCs) were isolated by Ficoll-Hypaque gradient separation and centrifuged for 20 min at 800*g*. The mononuclear cell-rich band was removed and resuspended in RPMI 1640 medium supplemented with l-glutamine, penicillin, streptomycin, and 10% fetal calf serum (FCS) and dispensed in microtiter 200 μl plates at a concentration of 1 × 10^6^ cells/well.

### Migration assay and confocal microscopy

Migration assay was performed in Thanswell chambers to evaluate PBMC migration toward DMSCs. PBMCs were added to the upper chamber in RPMI 1640 medium, and DMSCs were added to the lower chamber in DMEM/F12. PBMCs were cultured in the presence of DMSCs from normal donors (six females and six males) or patients (five females and seven males) with psoriasis with different PBMC/DMSC ratios (1 × 10^6^ cells/well:1 × 10^5^ cells/well, 10:1). After 24 h co-culture, live PBMCs were loaded with calcein AM (Dojindo Laboratories, Beijing, China) and the images were acquired with laser scanning confocal microscopy (LSCM, Olympus FV1200MPE, Japan). Calcein AM was excited with the 490 laser line and the emission recorded through a 515-nm band pass filter. The percentage of cell migrations was calculated on fluorescence-dying PBMCs versus the total PBMCs. Confocal microscopy and analysis of differences were obtained with software elaboration.

### Statistical analysis

SPSS 13.0 software (SPSS Inc., Chicago, IL, USA) was used to perform statistical analysis. All protein expressions and the percentage of migration were expressed as means ± SD. The statistical significance was determined using the unpaired two-tailed *t* test, and values of *P* < 0.05 were considered significant.

## Results

### Identification of DMSCs in vitro

**Fig. 1:**
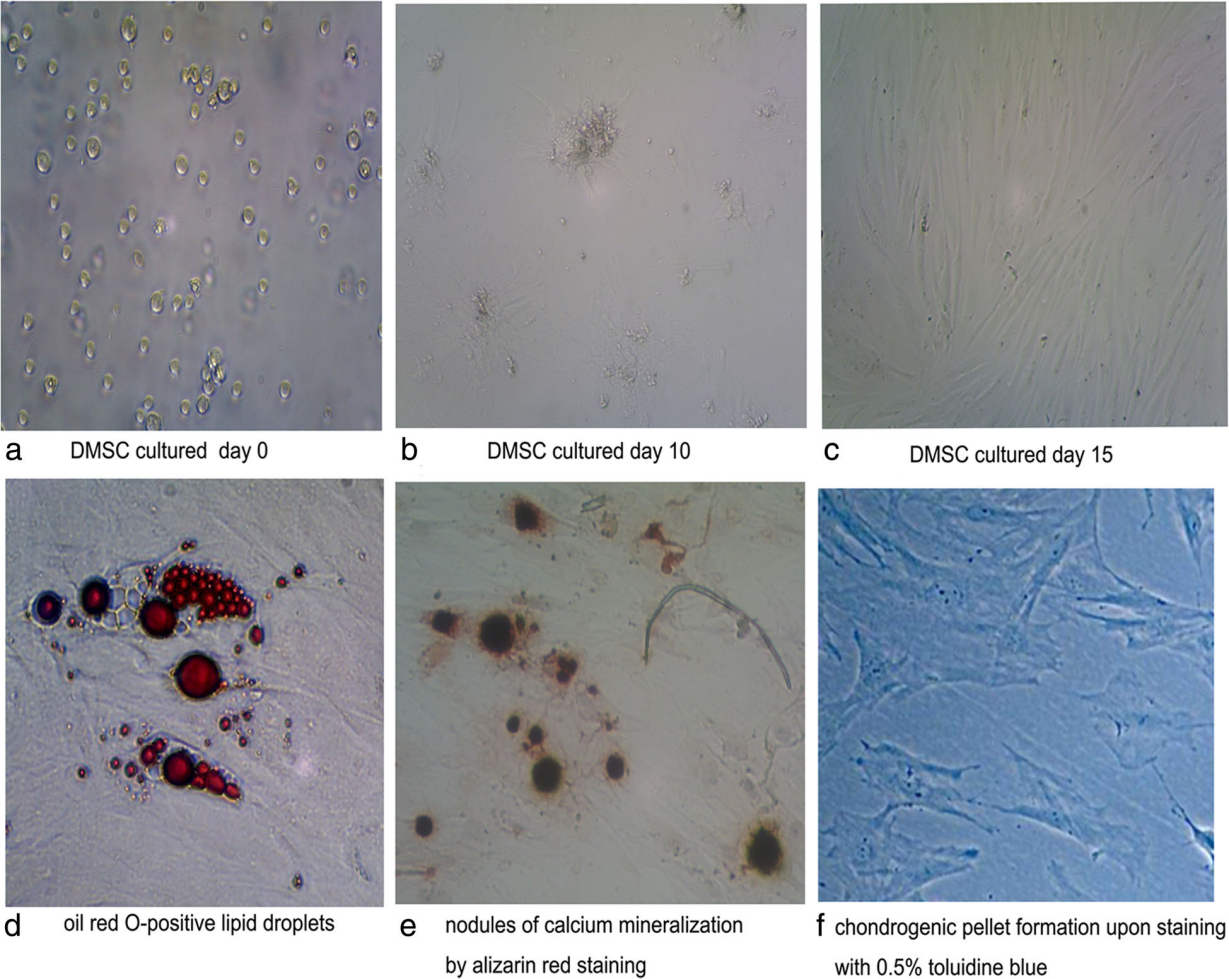
Morphological characteristics and differentiation ability of DMSCs in vitro (magnification × 40). DMSCs were cultured in vitro and differentiated to osteoblast, adipogenic, and chondrogenic cells. **a** The cultured cells at day 0, mainly fusocellular, triangular, and polygonal cells. **b** Cells at day 10, reached confluence. **c** Cells at day 15, DMSCs exhibited spindle-like morphologies. **d** Induced positive adipocyte, stained with Oil Red O. **e** Induced positive osteoblast nodules of calcium mineralization, stained with alizarin red. **f** Induced positive chondrogenic pellet formation, stained with 0.5% toluidine blue

Colonies of MSC-like cells began to appear in the culture flask 10 days after plating. After 2 weeks, the cells were washed and transferred to the fresh first-passage culture. When these cells grew to 90% confluence, they were reseeded into the fresh culture. At day 15, DMSCs exhibited spindle-like morphologies (Fig. 1a–c). Flow cytometry revealed that expression of the surface markers CD105, CD29, CD44, CD73, and CD90 was strongly positive, while that of CD14, CD45, and CD34 was negative. DMSCs were differentiated into osteoblast, adipogenic, and chondrogenic cells in the culture periods of 2 and 3 weeks, and almost all cells contained numerous Oil Red O-positive lipid droplets (Fig. 1d). Similarly, after DMSCs were cultured for 14 days, most of the cells showed aggregates or nodules of calcium mineralization by alizarin red staining (Fig. 1e) and were positive for chondrogenic pellet formation upon staining with 0.5% toluidine blue (Fig. 1f).

### mRNA expression of reference genes in psoriatic and normal DMSCs

**Fig. 2:**
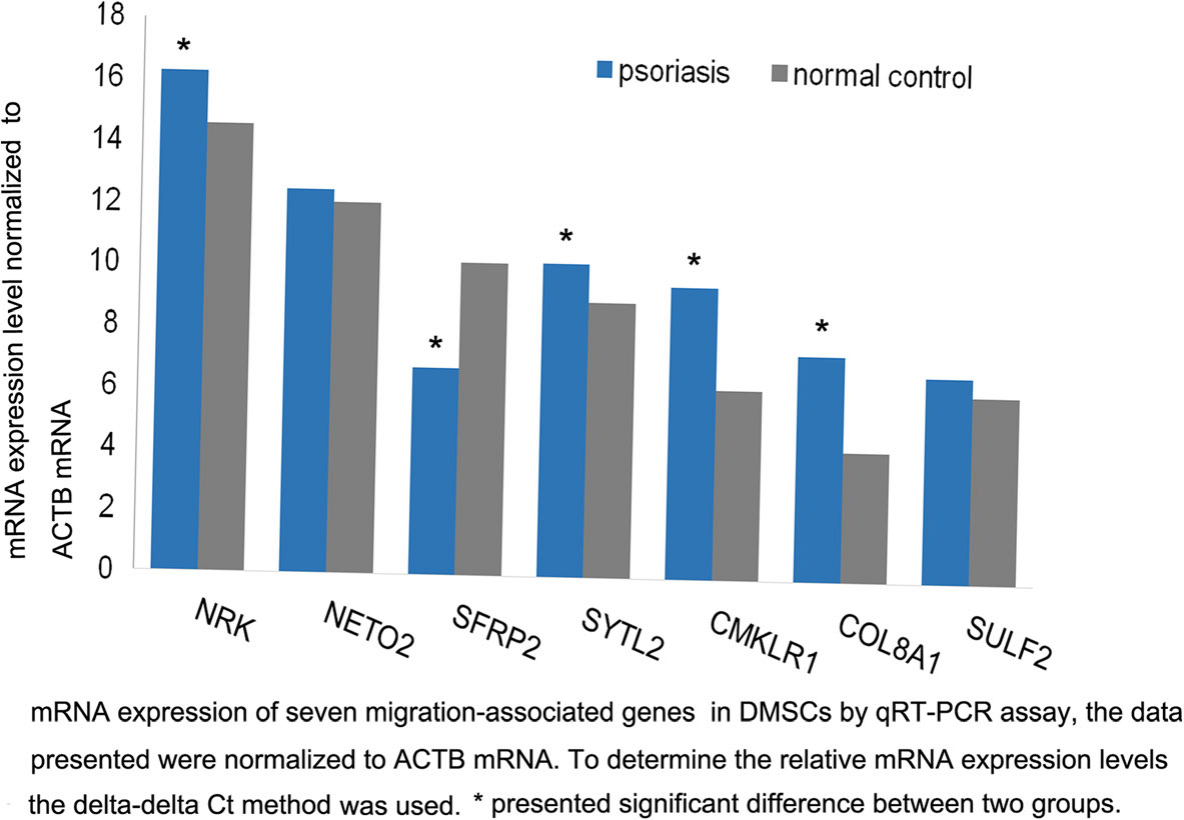
mRNA expression of seven reference genes including *CMKLR-1*, *COL8A-1*, *NETO-2*, *NRK*, *SFRP-2*, *SULF-2*, and *SYTL-2* in psoriatic and normal DMSCs. The data presented were normalized to *ACTB* mRNA. To determine the relative mRNA expression levels, we used the delta-delta Ct method (fold change). The expression of *SFRP-2*, *CMKLR-1*, *COL8A-1*, *NRK*, and *SYTL-2* differed significantly between the two groups (fold change of 2 or above)

To determine whether migration-associated genes in DMSCs affect immune cells, seven differentially expressed migration-associated genes in DMSCs by gene chips had been screened in our previous study [11]. In the present study, we verified those genes by qRT-PCR. Six of them were found to be upregulated and one to be downregulated in the psoriatic group compared with those in the control group. The expression of *SFRP-2* was shown to be downregulated by 0.09-fold in psoriatic DMSCs compared to that in controls, as assessed by qRT-PCR, while the expression levels of *CMKLR-1*, *COL8A-1*, *NETO-2*, *NRK*, *SYTL-2*, and *SULF-2* were upregulated, respectively, by 9.93-fold, 8.68-fold, 1.30-fold, 3.24-fold, 2.37-fold, 1.52-fold, in DMSCs with psoriasis compared to those in healthy controls (shown in Table 2). Another interesting aspect is that both *SFRP2* and *SULF2* belong to the Wnt signaling pathway. The mRNA expression levels of *SFRP-2*, *CMKLR-1*, *COL8A-1*, *NRK*, and *SYTL-2* differed significantly between two groups (Fig. 2).

**Table 2:** The mRNA expression of seven migration-associated genes in psoriatic and normal DMSCs

	Average ΔC_t_		ΔΔC_t_	2^-ΔΔ C^_t_
	Psoriasis	Control		
*CMKLR1*	9.50	6.19	3.31	9.93*
*SFRP2*	6.74	10.17	–3.43	0.09*
*NRK*	16.27	14.57	1.70	3.24*
*COL8A1*	7.36	4.24	3.12	8.68*
*NETO2*	12.46	12.08	0.38	1.30
*SULF2*	6.70	6.09	0.61	1.52
*SYTL2*	10.19	8.94	1.25	2.37*

Note: The data presented were normalized to ACTB mRNA

*Significant difference between the psoriatic group and normal group

### Protein expression of associated genes in psoriatic and normal DMSCs

**Fig. 3:**
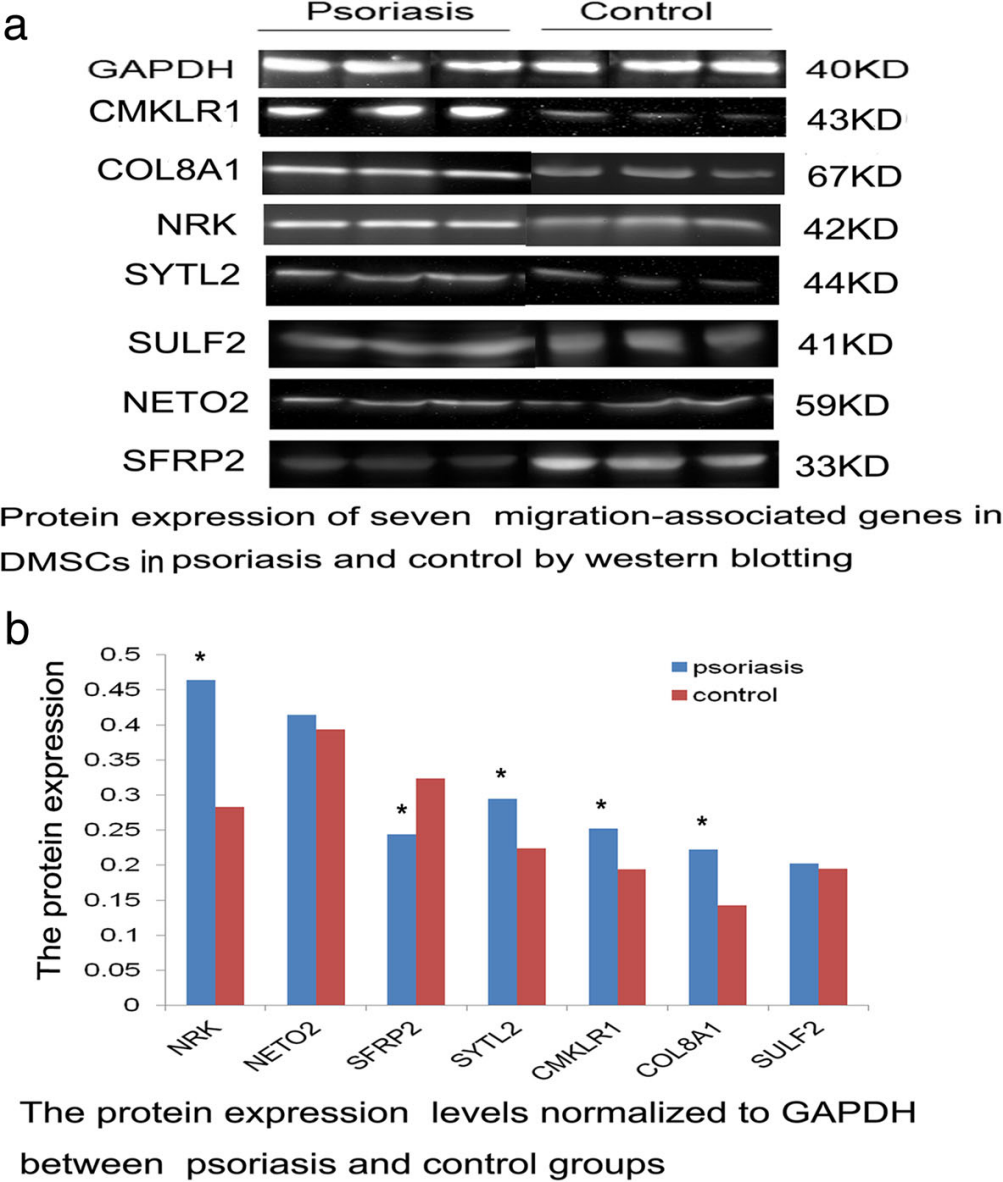
Protein expression of associated genes including *CMKLR-1*, *COL8A-1*, *NETO-2*, *NRK*, *SFRP-2*, *SULF-2*, and *SYTL-2* in psoriatic and normal DMSCs by western blot. **a** Molecular weights of 43 kDa, 67 kDa, 59 kDa, 42 kDa, 33 kDa, 41 kDa, and 44 kDa are specific to the respective *CMKLR-1*, *COL8A-1*, *NETO-2*, *NRK*, *SFRP-2*, *SULF-2*, and *SYTL-2* proteins. **b** Significant increases in protein expression of *CMKLR-1*, *COL8A-1*, *NRK*, and *SYTL-2* normalized to GAPDH were observed; however, *SFRP-2* was obviously decreased. Asterisk presented significant difference between the psoriatic group and normal group

Western blot assay showed the single bands corresponding to molecular weights of 43 kDa, 67 kDa, 59 kDa, 42 kDa, 33 kDa, 41 kDa, and 44 kDa and specific to the respective *CMKLR-1*, *COL8A-1*, *NETO-2*, *NRK*, *SFRP-2*, *SULF-2*, and *SYTL-2* proteins. We observed significant increases in protein expression of *CMKLR-1*, *COL8A-1*, *NRK*, and *SYTL-2* in DMSCs from patients with psoriasis compared with those from healthy donors, whereas the expression level of *SFRP-2* was obviously decreased (Fig. 3a, b).

### Evaluating DMSC/PBMC migration

**Fig. 4:**
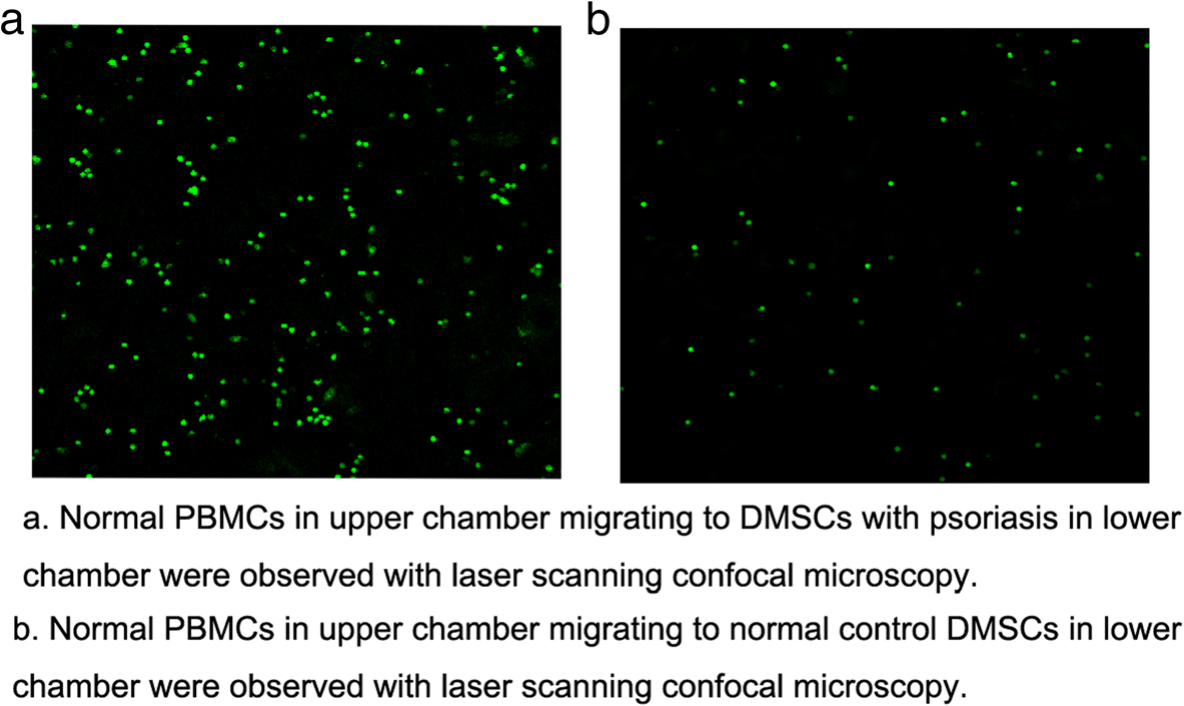
The normal PBMCs in the upper Thanswell chamber were observed to migrate into DMSCs from patients with psoriasis (**a**) and normal controls (**b**) in lower chamber after co-culture 24 h with laser scanning confocal microscopy in migration assay (magnification × 40)

The assay based on the Thanswell model was used to quantify cell migration. The results of the 24-h migration assay showed that in vitro the normal PBMC migration to the psoriatic DMSC group was a 6.3 ± 0.7-fold increase compared to the normal DMSCs group (*P* < 0.05, Table 3 and Fig. 4a, b) even though the cells did not have direct cell-to-cell contact.

**Table 3:** The migration quantity of normal PBMCs into DMSCs from psoriatic patients in comparison with normal controls with laser scanning confocal microscopy in migration assay

Group	Sample number	PBMCs migrated into lower chamber/the total PBMCs (%)	*P* value
Psoriatic DMSCs	12	76.5 ± 7.3	*P* = 0.003
Normal DMSCs	12	11.8 ± 0.9

## Discussion

Psoriasis is a common chronic inflammatory skin disease with a spectrum of clinical phenotypes and results from the interplay of genetic, environmental, and immunological factors. The pathogenesis of psoriasis involves dynamic interactions among multiple cell types and numerous cytokines in response to triggers, culminating in the disruption of skin immune homeostasis. One of the histopathological features of psoriasis is chronic inflammation densely infiltrated by T cells, DCs, NK cells, and neutrophils in the lesion, which migrate from the peripheral blood. In 2009, Valdimarsson et al. [12] proposed a modified schematic model to explain the potential role of CD4^+^ and CD8^+^ T cells in the development of psoriasis lesions. To date, several newly characterized T cell subpopulations and important innate immune cells are gradually noticed. Some studies have confirmed the involvement of T cell subsets in streptococcal tonsillitis and in psoriasis [13]. However, the precise pathogenetic mechanisms behind these cells are still unresolved in psoriasis. MSCs are multipotent non-hematopoietic precursors that can suppress proliferation and/or cytotoxic effect functions of distinct cell types of the innate and adaptive immune systems. The immunosuppressive capacity of MSCs is not constitutive, but is rather induced by crosstalk with cells of the immune system. Thus, the inflammatory environment—and in particular, the immune cells involved in each phase of an immune response—is likely to be critical triggers of this regulatory process. Many results have indicated that the inhibitory effect exerted by MSCs on immune cells can affect different aspects of activation and effect function, ranging from cytokine production to proliferation, differentiation, and migration.

In this study, to investigate whether DMSCs might participate in the pool of PBMCs, seven genes related to cell migration in psoriatic skin lesions were analyzed by qRT-PCR and western blotting. The results showed that the mRNA and protein levels of the psoriatic DMSC migration-related genes *SFRP2*, *CMKLR1*, *COL8A1*, *NRK*, and *SYTL2* were significant between the two groups. *CMKLR1*, *COL8A1*, *NRK*, and *SYTL2* were upregulated, and *SFRP2* was downregulated at both mRNA and protein levels. However, *NETO-2* and *SULF-2* were of no significant difference. A large data suggested that the canonical Wnt signaling pathway is activated in psoriasis [14], which has been shown to have fundamental roles in controlling cell proliferation, differentiation, cell adhesion, and movement [15]. *SFRP2* protein can bind Wnt proteins and inhibit Wnt signaling activity [16]. Chemerin is involved in the migration of DCs observed in inflamed tissues, and *CMKLR1* is a natural ligand of chemerin, controlling extracellular chemerin levels [17]. However, whether *CMKLR1* also mediates the migration of other cells is still poorly understood. *COL8A1*, an integral component of the basement membrane, is known to have the role of chemokine stromal cells and provide a substrate to enhance cell transfer [18]. Increased *COL8A1* expression is closely related to tumor cell proliferation and invasion. *NRK* is a Ser/Thr kinase and belongs to the germinal center kinase family, which suppresses the over-proliferation of mammary epithelial cells and mediates the apoptotic signaling triggered by tumor necrosis factor-α [19]. *SYTL2* is characterized as an effect protein for the Ras-related small GTPase [20]. Sung et al. [21] reported that the overexpression of *SYTL2* promoted metastatic potential, including increased migration and invasiveness in ovarian carcinoma cells. In the present study, the downregulation of *SFRP2* and overexpression of *CMKLR*, *COL8A*, *NRK*, and *SYTL2* reported herein revealed enhanced migratory and invasive potential abilities from DMSCs to other cells in psoriasis, which may be beneficial to the immunomodulatory properties of MSCs. It is necessary to investigate the exact mechanism for further study in the future.

To our knowledge, no previous publications have reported the influence of the DMSCs on PBMC migration. Our aim in this study was to establish whether DMSCs had a positive influence on PBMC migration. In the Thanswell chamber analysis, DMSCs of patients with psoriasis were found to stimulate normal PBMC migration. The results of the 24-h migration assay clearly demonstrate that DMSCs can induce PBMC motility and increase both the total number of cell migration as well as the rate of cell movement. Additionally, the migration assay showed that no direct cell-to-cell contact was required as the directed cell movement was observed when MSCs were in the lower chamber separated from the PBMCs in the upper chamber. The migration of PBMCs is known to be regulated by a variety of cytokines. Future work should investigate the individual chemotactic factor or factors present in DBMCs in order to harness the full potential of the directed cell migration property.

## Conclusion

Our findings provide novel evidence for DMSCs as an epigenetically regulated pro-migration factor that is associated with disorder of immune in psoriasis. Although further investigations are required to understand the comprehensive molecular mechanisms of abovementioned *CMKLR*, *COL8A*, *NRK*, *SYTL2*, and *SFRP2* genes, this study suggests the potential use of *CMKLR*, *COL8A*, *NRK*, *SYTL2*, and *SFRP2* as a therapeutic target for psoriasis in the future.

## Additional file

Additional file 1: Clinical information of samples with psoriasis. The clinical information included the gender, age, disease history and psoriasis area and severity index (PASI) of patients. (XLSX 10 kb)

## Abbreviations

bFGF: Basic fibroblast growth factor
*CMKLR-1*: Chemokine-like receptor-1
*COL8A-1*: Collagen type VIII alpha1
DCs: Dendritic cells
DMEM: Dulbecco’s modified Eagle’s medium
DMSCs: Dermal mesenchymal stem cells
EGF: Epidermal growth factor
FBS: Fetal bovine serum
FCS: Fetal calf serum
GAPDH: Glyceraldehyde-phosphate dehydrogenase
HGF: Hepatocyte growth factor
IL-1: Interleukin-1
LSCM: Laser scanning confocal microscopy
MSCs: Mesenchymal stem cells
*NETO-2*: Neuropilin and tolloid-like 2
NK: Natural killer
*NRK*: Nik-related kinase
PBMCs: Peripheral blood-derived mononuclear cells
qRT-PCR: Quantitative real-time reverse transcription PCR
SCF: Stem cell factor
SDS-PAGE: Sodium dodecyl sulfate-polyacrylamide gel electrophoresis
*SFRP*: Secreted frizzled-related protein
*SULF-2*: Sulfate 6-*O*-endosulfatase 2
*SYTL-2*: Synaptotagmin-like protein 2
Th1: Help T cells
Tregs: Regulatory T cells
